# Impact of Hearing Loss Severity on Hearing Aid Benefit Among Adult Users

**DOI:** 10.3390/healthcare12232450

**Published:** 2024-12-05

**Authors:** Marlena Ziemska-Gorczyca, Karolina Dżaman, Ireneusz Kantor

**Affiliations:** Department of Otolaryngology, Centre of Postgraduate Medical Education, Marymoncka 99/103, 01-813 Warsaw, Poland; marlena.z.gorczyca@gmail.com (M.Z.-G.); ireneusz.kantor@gmail.com (I.K.)

**Keywords:** hearing aid, APHAB (abbreviated profile of hearing aid benefit), assessment of hearing-aid benefit, hearing loss, evaluation of hearing-aid use

## Abstract

Background: Hearing loss (HL) among older adults is a major global health concern. Hearing aids (HAs) offer an effective solution to manage HL and enhance the quality of life. However, the adoption and the consistent use of HAs remain low, making non-use a significant barrier to successful audiological rehabilitation. The aim of the study was to assess the benefit of HAs among patients with different degrees of HL and to determine the profiles of patients who have the least benefit from HAs. Methods: the HA benefits were assessed by using the Abbreviated Profile of Hearing Aid Benefit (APHAB) questionnaire. Participants were assigned to the study groups based on the pure-tone audiometry. This paper presents the results obtained by using HAs in various listening environments among 167 patients. Results: The majority of individuals benefited from HAs in a noisy environment while a reverberant environment provided the lowest benefit. It was observed that the degree of HL had a statistically significant impact on the benefits of HAs in terms of the communication ease, the reverberation, the background noise, and the global score. A moderately positive correlation was observed between the unaided APHAB and the HL degree. The subjects’ APHAB scores ranged from the 50th to the 65th percentile. Additionally, women had a significantly better improvement than men. Conclusions: HAs improved communication in everyday life situations among 91.6% of HA users. The degree of HL influences APHAB scores. Patients with a severe degree of HL achieved the greatest APHAB scores while male patients with mild HL received the lowest benefits of HAs. Both HL and the age, gender, and HA type are factors that also play important roles. The APHAB questionnaire is a reliable screening test for patients with hearing difficulties.

## 1. Introduction

Hearing loss (HL) is one of the most common disabilities among older adults, with an estimated one in four people over the age of 60 being affected by it [1]. According to the 2014 TNS Poland report (Polish Public Opinion Researching Agency), 77% of individuals over the age of 60 years reported experiencing hearing problems [2].

Individuals with HL frequently struggle to learn and are more likely to be unemployed [1]. Not only does this condition impair their general well-being and mental abilities, but it also exacerbates feelings of anxiety and depression [3,4].

Among the various factors contributing to dementia, HL is one of the most significant [5,6]. A multicenter, randomized controlled trial concluded that a hearing intervention could prevent older adults being at higher risk of cognitive decline from developing dementia over a three-year period [6].

Despite the noted benefits of hearing aids (HAs) for speech perception, cognition, and psychosocial health [4,5], many hearing-impaired people do not decide to use HAs giving reasons including such external factors as the high price of HAs or that doctors did not suggest HAs to them [7]. However, the patients’ family members also report that the negative attitude towards the device results from a sense of stigmatization and the patients’ beliefs that there is no need for assistance with the device [7]. What is more, former HA users report barriers related to comfort and ineffectiveness of HAs as well [7].

The main purpose of HAs is to improve communication between a hearing-impaired person and the environment, and consequently, to increase their quality of life [4,7,8]. From an audiological point of view, the important aspect is to prevent deprivation (retrogradation) processes in the central structures of the auditory pathway, i.e., a lack of speech understanding [9]. However, qualifying patients for HA treatment often met with their reluctance and prejudice, thus a holistic approach focused on showing the patient the most important goal, which is to improve communication and reduce social isolation, is crucial to encourage the patient to use the device [4].

HAs are one of the most effective methods of hearing rehabilitation. There are many types of HAs available on the market and various questionnaires used to assess their effectiveness, which play a key role in audiological assessments.

Along with tests for pure tones, loudness discomfort, and speech audiometry, surveys offer an individual subjective perspective, helping the clinician gain a more complete understanding of the patient’s hearing difficulties. Based on Guidelines for the Evaluation of Hearing Aid Fitting, questionnaires are one of eight points recommended for use during the HA fitting process [10]. Thus, surveys can be a convenient and reliable tool for monitoring the HA fitting process.

There are various audiological measures used to assess the effectiveness of HAs, but one of the most reliable and the most commonly used scales is The Abbreviated Profile of Hearing Aid Benefit (APHAB) [11,12]. The APHAB is divided into four subscales with six items each:

1. Ease of communication scale (EC)—evaluates communication effort in a simple listening situation without the background noise
2. Background noise scale (BN)—evaluates speech understanding with the environmental competing noise
3. Reverberation scale (RV)—evaluates hearing in large rooms with echo or reverberation
4. Aversiveness of sounds scale (AV)—evaluates negative reactions to loud environmental sounds.

The first three subscales (EC, RV, and BN) measure speech understanding in a variety of everyday listening situations, and the fourth subscale (AV) includes reactions to loud environmental sounds. Positive values in the comparison of unaided to aided situations suggest the benefit of HAs. For example, the global score benefit of 44% means that when using a HA, communication difficulties are reduced by 44%. When the aided subscale scores differ by 22 percentage points or more, it can be assumed that the fitting is significantly more successful [12]. As expected, the scores reflecting differences in the AV subscale tended to show negative values following amplification. This indicates an increased incidence of problems related to discomfort caused by environmental sounds when using HAs [13].

Over the years, scientists have observed that new technologies in HAs have not significantly improved EC, RV, and BN benefits [13]. However, for the AV subscale, the capabilities of current HAs resulted in fewer negative reactions to environmental sounds compared to mainly linear HAs without the digital noise reduction, addressing a common complaint that HAs make many everyday sounds objectionably loud [13]. Furthermore, the APHAB questionnaire can be also used as a pre-qualification evaluation for HAs, helping to assess the hearing problems in everyday situations. Only few researchers compared the APHAB score to pure-tone audiometry (PTA) and brainstem auditory evoked potentials (BAEPs). It was concluded that correlations were observed only between the APHAB and PTA [14]. However, there is still limited knowledge regarding the relationship between HA benefit in the APHAB and audiological measures. To our knowledge, this is the first study comparing pure-tone audiometry results with the APHAB questionnaire for the Polish population. 

The aim of this study was to assess the benefit of HAs in patients with different degrees of hearing loss based on a previously used Polish translation of the APHAB questionnaire [15,16] and pure-tone audiometry. In addition, we aimed to determine the profile of the patient who received the least benefit from HAs, which may be useful for ENT specialists, audiologists, and HA technicians during the HA qualification and fitting process.

## 2. Materials and Methods

### 2.1. Study Design and Participants

The study included 167 patients of the Outpatient Clinic of Audiology in the Mazovian Bródno Hospital, diagnosed with various degrees of hearing loss (mild, moderate, severe, and profound). The data were collected over a period of 3 years (2018–2020) and were based on the audiological clinical visit outcome.

All of the patients were examined physically by an audiologist and were fitted with HAs either in the canal (ITC) or behind the ear (BTE) as a part of clinical practice for at least 3 weeks. HAs used in observation were manufactured by various companies. All of the participants provided consent for the use of their clinical data (age, gender, APHAB results, PTA results of pure-tone audiometry, and type of HA) in the study. All patients were evaluated both with the APHAB questionnaire and PTA.

### 2.2. The Inclusion Criteria

1. Adults diagnosed with bilateral symmetrical sensorineural HL with a <15 dB difference in PTA (average of 500, 1000, 2000, and 4000 Hz) between the ears, and a <10 dB difference between air and bone conduction thresholds, confirmed via PTA.
2. Patients who came for a HA replacement or for first-time HA selection.
3. Individuals who consented to participate and complete the APHAB questionnaire.

### 2.3. The Exclusion Criteria

1. Patients with incomplete audiological or APHAB data.
2. Individuals who failed to use their HAs consistently during the study period.
3. Subjects with asymmetric or conductive HL.

Patients with asymmetric and conductive hearing loss were excluded to standardize the study group in terms of hearing-loss type in order to reduce the number of factors that would affect the results of the statistical analysis.

### 2.4. Questionnaire

HA benefit was measured on the basis of the APHAB questionnaire. The APHAB questionnaire measures subjective hearing loss before and after HA fitting in four situations. The first part of the APHAB questionnaire (APHAB u) can be used regardless of HA use. It consists of 24 items that represent a specific scenario. The patients are required to assess how frequently each statement reflects their everyday experience. Individuals must select the most appropriate response from a range of seven descriptive options, each associated with a specific percentage from never (1%) to always (99%) [13,17,18,19]. The APHAB questionnaire is completed by providing two responses to each item: “without my HA” and “with my HA”. In our study, the benefit was calculated by subtracting the mean percentages of the unaided and aided parts of the APHAB subscales.

The APHAB questionnaire was administered to evaluate the perceived benefit of HAs in different environments (e.g., the ease of communication, reverberations, the background noise, and the global score (GS) benefit). The unaided part of APHAB questionnaire was completed during the qualification appointment of the patient and the aided part of the APHAB questionnaire was completed after the patient’s trial use of a HA.

Hearing-loss severity was measured by using PTA to classify patients into subgroups (mild, moderate, severe, and profound hearing loss). PTA data were used to correlate the degree of hearing loss with perceived HA benefits.

### 2.5. Ethical Considerations

The guidelines set by the Declaration of Helsinki were followed in the study. Since the study is non-invasive, it did not require ethical committee approval. However, the use of the obtained data was preceded by obtaining informed consent from all of the abovementioned patients.

### 2.6. Statistical Analysis

The relationship between the degree of hearing loss and the APHAB scores was analyzed by the use of R Pearson correlation coefficients and the Kruskal–Wallis test. ANOVA and repeated-measures Student’s *t*-tests were used to compare the HA benefits between ITC and BTE users and between male and female patients. Statistical tests were performed in R statistical software (version 4.2.3) and statistical significance was set at *p* < 0.05.

## 3. Results

A total of 167 patients (90 men—53.9%, 77 women—46.1%) with different degrees of hearing loss participated in the study. The average age was 68.9 years (SD 13.8; range 23–94 years old). There was no statistically significant difference between the right- and left-ear PTA results. The average pure-tone thresholds for the 500, 1000, 2000, and 4000 Hz frequencies were 56.0 dB (SD 18.3) for the right ear and 56.6 dB (SD 17.2) for the left ear. Patients with hearing loss were divided into four groups based on the degree of HL according to the WHO criteria: mild (21–40 dB)—26 patients, moderate (41–60 dB)—87 patients, severe (61–80 dB)—38 patients, and profound (≥81 dB)—16 patients [20]. The structure and characteristics of the study groups are presented in Table 1.

The largest study group comprised participants with moderate HL—52.1% of all patients. Severe or profound HL was diagnosed in one in three patients and this group was dominated by women.

Patients with severe HL achieved the best GS benefits with HAs showing statistically significant differences in their APHAB results before and after using the device (*p* = 0.005).

The lowest GS benefits from HAs were noticed in patients with a mild degree of HL; the benefits were also statistically significant (*p* = 0.035). The youngest group achieved the lowest GS benefits.

The results of the APHAB questionnaire are presented in Table 2. A positive GS benefit from HA was achieved by 91.6% (153) of patients.

Comparing unaided to aided conditions, a significant reduction in the reported frequency of communication problems in three APHAB subscales: EC, BN, and RV, and the total GS APHAB score (*p* < 0.01), was noticed (Table 2). Patients reported, on average, 40% fewer speech-comprehension problems in various conditions. In the background noise conditions, the reductions were the highest and they amounted to 44.2%. Comparing BN benefit to other APHAB subscales in repeated Student’s *t*-test, there were significant differences in all cases (*p* < 0.05). Additionally, there was a significant increase in the reported frequency of negative reactions or aversions to environmental sounds in the unaided condition compared to the aided condition (AV subscale) (*p* < 0.01). A weak negative correlation between HL degree and AV benefit was found while using the Pearson correlation (Pearson correlation −0.18, *p*-value 0.02).

In the case of EC, BN, AV, and GS benefits, correlations between HL degree were positive but very weak. The results of the Pearson correlation are presented in Table 3.

The Pearson correlation between the degree of HL and unaided subscale scores was also performed, and what is more, it revealed that the degree of HL had a moderately positive correlation with most of the unaided scores, particularly with GS unaided (0.37) and BN unaided (0.37) (Table 4).

**Figure 1:**
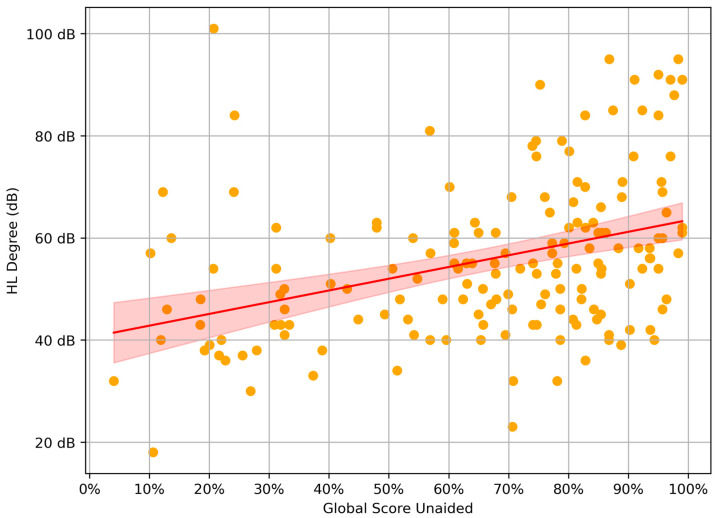
The relationship between global score unaided and the degree of HL.

The relationship between GS unaided and the HL degree can be visualized in Figure 1.

**Figure 2:**
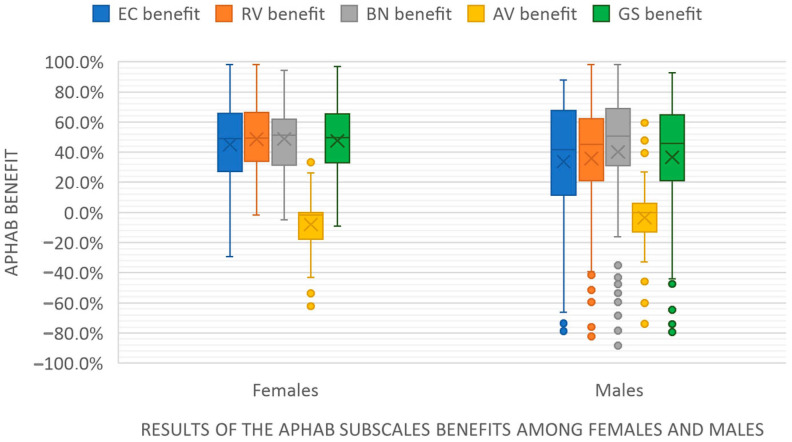
Comparison of APHAB subscale benefits by gender. Legend: EC—ease of communication, RV—reverberation, BN—background noise, AV—aversiveness, GS—global score.

Women had statistically significantly better results in terms of EC, RV, and GS benefit scores than men (*p* < 0.05); the results are presented in Figure 2 and Table 5. Females reported significantly less communication problems without background noise and in reverberant environments compared to men. With respect to the audiometric data, women were more likely to demonstrate severe hearing loss than men. 

**Figure 3:**
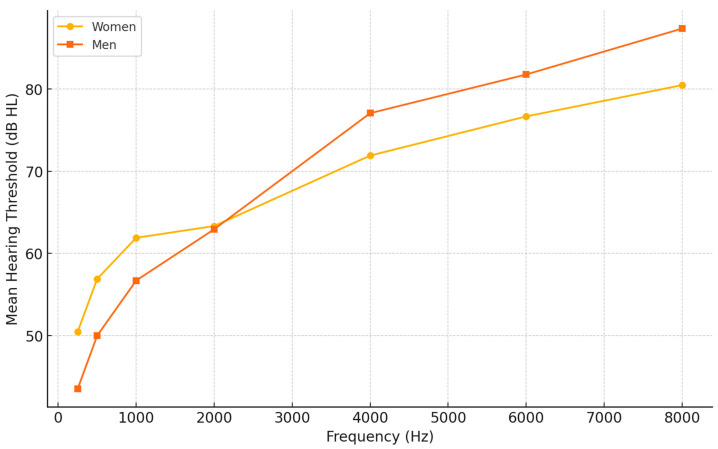
Comparison of audiograms by gender.

The analysis revealed that in the group with severe HL, men had greater degree of hearing loss at high frequencies than women (*p* < 0.05). The audiograms are presented in Figure 3.

Comparing four degrees of HL (according to the WHO criteria) with APHAB results in the Kruskal–Wallis test, we observed the impact of the hearing impairment level on the final benefit from HAs in terms of EC benefit, RV benefit, BN benefit, and GS benefit (Table 6). Patients with a severe degree of HL achieved the greatest benefits in EC, RV, BN, and GS.

**Figure 4:**
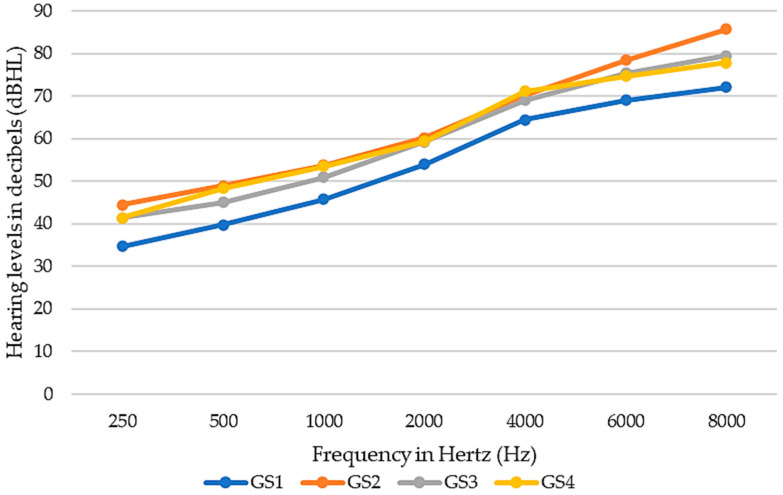
Results of pure-tone threshold audiometry in global score (GS) benefit groups.

Additionally, we divided patients into four equal groups according to increasing GS benefit. One-way ANOVA and post-hoc analysis revealed that the GS1 group (group with the lowest benefit from HA) was significantly younger and had a significantly lesser degree of HL than the other GS benefit groups (Table 7). Such a correlation was not noticed in the other groups (GS 2–4) (Table 7). The other groups, irrespective of GS in the APHAB questionnaire, had similar PTA results (Figure 4). Moreover, the Pearson correlation was performed and it was revealed that the benefit of GS had an insignificant correlation with the average age of the patient (r = 0.02; *p*-value = 0.77).

The patients included in the study were BTE and ITC users. The ITC group consisted of 20 patients. The BTE group included 147 patients. In both the BTE and ITC groups, patients with moderate hearing loss constituted the largest groups. Detailed data are presented in Table 8.

Statistical analysis revealed that the type of HA might influence the EC and BN benefits, while the other benefits did not appear to be dependent on HA type based on a conventional alpha level of 0.05. The patients with ITC achieved better EC and BN improvements than BTE users and the difference was statistically significant. The results presenting the distribution of HA types are shown in Table 9.

## 4. Discussion

In this study, statistical analysis revealed that the degree of HL had moderately positive correlations with unaided EC, RV, BN, and GS scores. Consequently, as HL worsened, patients reported more significant communication problems in various environmental conditions. This observation is in the line with the study of Löhler et al., where the sensitivity and specificity of the APHAB questionnaire as the method of detecting HL of at least 25 dB were calculated to be 0.85 and 0.81 [21]. Some authors also showed that the unaided part of APHAB questionnaire referring to a person’s experience without HA can be useful in primary audiological diagnoses, regardless of the subsequent evaluation of HA fitting [22,23]. We also confirmed the lack of association between PTA and the AV unaided subscale, which is consistent with the findings of a previous study [24]. We suspected that the AV unaided subscale was not related to PTA because the AV subscale relates to the unpleasantness of environmental sounds, whereas PTA examines the detection of soft sounds [24,25].

Based on the Kruskal–Wallis test, the degree of HL had an impact on the APHAB benefit in the EC, RV, BN, and GS subscales; however, no significant correlation was found in the Pearson correlation test. This is an understandable conclusion due to the fact that the benefit from a HA is determined by many factors and therefore the level of HL has an impact on the benefit from the HA, but it is not a linear correlation. Other authors have come to the similar conclusion in this matter [25]. 

Based on the study of Cox et al., the 50th percentile of the APHAB subscale benefits for contemporary HAs are EC = 38, BN = 34, RV = 33, AV = −13, and GS = 35 [13]. The scores for the current study were between the 50th and 65th percentiles. These results suggest that the present study group achieved slightly greater benefits from HAs than average HA users. This may reflect that the norms for the APHAB subscale benefits were updated in 2010, while the technology of HAs is constantly being improved, and consequently, patients achieved greater benefits. Results of APHAB subscale benefits presented in this paper are significantly better than for another Polish group of patients in the study of Pietruszewska et al. (EC benefit 38.9% vs. 16%, BN benefit 44.2% vs. 16%) [26]. Such large differences may result from the profiles of the study groups. In the group studied by Pietruszewska et al., there were long-term HA users, while in the presented study group, most patients were being qualified for a HA for the first time.

Our findings suggest that the greatest GS benefit from HAs was achieved in patients with severe HL. In contrast, those with mild HL had the least benefit from using a HA. The GS1 group consisted of those with the lowest GS benefits, who had the lowest degree of HL (Figure 2). An explanation for these results may be the fact that patients with mild HL functioned relatively well without a HA, and consequently achieved low results with the APHAB questionnaire without a HA. Similar results have been reported by other researchers [27].

HAs produced the greatest improvement in noisy environments (BN subscale). In the study of Poremski et al., patients achieved the worst benefit during conversation in silent rooms (EC subscale); the subjects perceived a greater benefit from the HA in noisy environments compared to the quiet ones [27]. Similarly, in another study, HA users performed worse in quiet environments compared to noisy ones [28]. This is a very important positive finding for patients with HL, who mostly have problems with communication in noisy conditions. The fact that HAs improve their users’ communication to a lesser extent in quiet conditions than with background noise may be due to the fact that patients with sensorineural hearing loss more often have difficulties with communication in quiet environments. Persons with sensorineural hearing loss receive significantly less benefit from HAs because the problem lies in the correct conduction and processing of received sounds in the nervous system [29].

Untreated HL has been shown to lead to a deterioration in cortical and cognitive function [30,31]. Therefore, an early decision to use a HA offers a better chance of obtaining greater benefits from using a HA due to improved auditory plasticity. Patients aged 60 years and older attain less benefit [32].

Based on our results, women achieved significantly better benefits in the EC, RV, and GS subscales than men. It is worth noting that men, despite having a similar degree of HL, often have worse HL in high tones (≥2000 Hz) when compared to women, which we also noticed in our study (Figure 3). The greater degree of high-frequency HL in men may be due to their more frequent exposure to excessive noises, which primarily cause acoustic trauma [33]. The selection of HA in patients who have significantly greater HL in the frequency range above 1000 Hz than in the low frequencies is much more difficult. Generally, patients with high-frequency HL benefited less from HAs due to the limited capacity of the HAs. The other cause of the fact that women achieved greater benefits from HAs is that the nonregular use of HAs is more prevalent in men [33]. Regular use and a longer daily duration of HA use increases the chances of higher satisfaction from a HA.

The weakest gains were made on the aversiveness scale, where only 29% of participants scored positively. Nevertheless, these results are between the 65th and 80th percentiles compared to Cox et al.’s norms [13]. Therefore, when fitting a HA, it is crucial to meticulously select acoustic settings that affect the perception of unpleasant sounds. On the other hand, some authors have stated that clinicians may not prioritize issues related to the loudness of amplified sounds; as such, problems are less common compared to the challenges faced by patients in reverberant and noisy environments [13].

Statistical analysis showed that the type of HA had an impact on APHAB subscale benefits. ITC users achieved statistically significantly greater benefits on the EC and BN subscales than BTE users. This contradicts the results of previous studies conducted a long time ago, in which no differences between the two types of HA were observed [34]. Importantly, as mentioned above, Cox et al. determined that a 22% difference between the APHAB results for two different devices is considered significant. In our study, the differences observed between the groups were consistently less than this 22% threshold across all APHAB subgroups. Thus, while we identified some differences, these do not meet the established cutoff for significance as defined by Cox et al. In conclusion, the observed differences, although present, are not meaningful in the context of this significance criterion. A recent article comparing the effectiveness of BTE and completely-in-the-canal (CIC) devices concluded that there were not significant differences in the APHAB results between BTE and CIC users [28].

The limitation of this study can be seen as the insufficient sample size of patients as the calculated required total sample size is 180 individuals. Due to the fact that this is a single-center study, it was difficult to obtain such a study group. We decided to undertake the analysis, because the number of patients qualified for the study was only slightly below the required number. Additionally, there was a significant imbalance in the sample sizes between the ITC and BTE groups, which could affect the reliability of the results. Moreover, the duration of HA use during the day was not registered in this study. Finally, some data of the study come from artificially created groups, which may further limit the study’s generalizability.

## 5. Conclusions

In conclusion, more than 90% of HA users have benefited positively from GS in the APHAB subscale. This is a very important conclusion that helps to raise awareness among patients who have doubts about whether to choose a hearing aid. Patients with HL are increasingly benefiting from the use of a HA over the years. Not only the degree of HL, but also age, gender, and the type of HA influence the benefits of HAs. Patients with severe HL benefited the most from HAs. Among different communication environments, communication with the background noise was improved at the highest level by HAs. Women achieved significantly greater benefits from a HA than men. High-frequency hearing loss reduces the benefits of HAs. The APHAB questionnaire can help to measure the impact of hearing loss on a person’s ability and the improvement that comes with using a HA. In light of our results, which indicate the effectiveness of using the APHAB questionnaire to assess a scale of communication difficulties among patients, it seems reasonable to consider its widespread use in general practitioners’ offices for screening tests as well. 

## Figures and Tables

**Table 1:** Characteristics of HL groups.

Degree of HL	Age in Years (SD)	Count	Females	Males	Average HL in Decibels (SD)	Global Score Benefit (SD)
Mild	62.0 (12.4)	26 (15.6%)	11 (42.3%)	15 (57.7%)	35.8 (5.49)	29.2% (31.22)
Moderate	69.8 (13.24)	87 (52.1%)	35 (40.2%)	52 (59.8%)	50.6 (5.76)	42.8% (36.67)
Severe	71.5 (13.17)	38 (22.8%)	21 (55.3%)	17 (44.7%)	67.6 (5.88)	47.6% (29.44)
Profound	68.2 (18.67)	16 (9.5%)	10 (62.5%)	6 (37.5%)	88.8 (5.38)	42.3% (29.43)

**Table 2:** Results of APHAB questionnaire with and without hearing aid.

	EC	RV	BN	AV	GS
APHAB unaided	59.5%	68.3%	71.0%	16.4%	66.3%
APHAB aided	20.6%	26.4%	26.8%	22.0%	24.6%
Benefit	38.9%	41.9%	44.2%	−5.58%	41.7%
*p*-Value	<0.01	<0.01	<0.01	<0.01	<0.01

Legend: EC—ease of communication, RV—reverberation, BN—background noise, AV—aversiveness, GS—global score.

**Table 3:** The results of Pearson correlation between APHAB scores and HL degree.

APHAB	Pearson Correlation with HL Degree	*p*-Value
EC benefit	0.15	0.05
RV benefit	0.11	0.17
BN benefit	0.09	0.23
AV benefit	−0.18	0.02
GS benefit	0.12	0.11

Legend: EC—ease of communication, RV—reverberation, BN—background noise, AV—aversiveness, GS—global score, HL—hearing loss.

**Table 4:** The results of Pearson correlation between unaided subscales of APHAB questionnaire and HL degree.

APHAB Unaided	Pearson Correlation with HL Degree	*p*-Value
EC unaided	0.36	<0.01
RV unaided	0.35	<0.01
BN unaided	0.37	<0.01
AV unaided	−0.09	0.25
GS unaided	0.37	<0.01

Legend: EC—ease of communication, RV—reverberation, BN—background noise, AV—aversiveness, GS—global score, HL—hearing loss.

**Table 5:** Comparison of APHAB scores by gender.

APHAB	*t*-Statistic	*p*-Value	Female Mean (SD)	Male Mean (SD)
EC benefit	2.09	0.04	45.0% (27.7)	33.8% (41.5)
RV benefit	2.56	0.01	48.8% (22.4)	35.9% (40.9)
BN benefit	1.69	0.09	48.9% (22.1)	40.2% (42.8)
AV benefit	−1.37	0.17	−7.9% (19.9)	−3.5% (22.4)
GS benefit	2.20	0.03	47.6% (22.5)	36.7% (40.7)

Legend: EC—ease of communication, RV—reverberation, BN—background noise, AV—aversiveness, GS—global score, SD—standard deviation.

**Table 6:** The Kruskal–Wallis test results—comparison of the degree of HL with APHAB subscale benefits.

	EC Benefit (SD)	RV Benefit (SD)	BN Benefit (SD)	AV Benefit (SD)	GS Benefit (SD)
Mild	24.93% (31.27)	29.13% (33.14)	33.43% (32.19)	−3.72% (12.2)	29.2% (31.22)
Moderate	39.73% (38.63)	43.09% (37.16)	45.31% (38.17)	−2.47% (22.12)	42.8% (36.67)
Severe	44.77% (32.91)	47.47% (28.55)	50.36% (29.45)	−9.1% (20.02)	47.6% (29.44)
Profound	43.55% (33.43)	42.47% (28.62)	41.01% (31.82)	−17.11% (27.33)	42.3% (29.43)
Statistic	8.37	8.09	8.83	5.15	9.07
*p*-Value	0.04	0.04	0.03	0.16	0.03

**Table 7:** Characteristics of groups divided on the basis of the global score benefits.

GS Benefit Group	GS Benefit Mean (SD)	Age in Years (SD)	HL in Decibels (SD)
GS1	−3.2% (33.4)	65.2 (11.9)	50.4 (15.8)
GS2	39.7% (5.5)	72.8 (14.3)	58.1 (16.2)
GS3	55.3% (5.1)	72.0 (10.9)	58.0 (16.2)
GS4	75.3% (8.2)	65.6 (16.5)	56.5 (13.3)
*p*-Value		0.01	0.005

Legend: GS—global score, HL—average hearing loss in decibels.

**Table 8:** Distribution of BTE and ITC users in the HL groups.

Degree of HL	ITC Group	BTE Group
Mild	10% (2 patients)	16.3% (24 patients)
Moderate	45% (9 patients)	53.1% (78 patients)
Severe	35% (7 patients)	21.1% (31 patients)
Profound	10% (2 patients)	9.5% (14 patients)

Legend: HL—hearing loss, ITC—in-the-canal hearing aid, BTE—behind-the-ear hearing aid.

**Table 9:** Distribution of reported HA types and APHAB subscale benefits.

Type of HA	GS Benefit	EC Benefit	BN Benefit	RV Benefit	AV Benefit
ITC	52%	58%	58%	50%	−2%
BTE	40%	37%	42%	41%	−6%
*p*-Value	0.06	**0.02**	**0.04**	0.39	0.57

Legend: HA—hearing aid, ITC—in-the-canal hearing aid, BTE—behind-the-ear hearing aid, GS—global score, EC—ease of communication, BN—background noise, RV—reverberation, AV—aversiveness. The bold means the difference in these subscales is statistically significant *p* < 0.05.

## Data Availability

The original contributions presented in the study are included in the article; further inquiries can be directed to the corresponding author.
